# Natural Killer Subset Changes and Vascular Endothelial Growth Factor‐A Plasma Profile in Progressive Supranuclear Palsy: The NKscape Study

**DOI:** 10.1002/mds.70273

**Published:** 2026-03-17

**Authors:** Marina Picillo, Cristina Basile, Francesco Montella, Valentina Lopardo, Mariangela Orlando, Roberta Tarallo, Domenico Palumbo, Viola Melone, Maria Francesca Tepedino, Paolo Barone, Annibale Alessandro Puca, Elena Ciaglia

**Affiliations:** ^1^ Neuroscience Section, Department of Medicine and Surgery “Scuola Medica Salernitana” University of Salerno Fisciano Italy; ^2^ IRCCS SYNLAB SDN Naples Italy; ^3^ School of Specialization in Clinical Pathology and Clinical Biochemistry University of Salerno Fisciano Italy; ^4^ Department of Medicine, Surgery and Dentistry “Scuola Medica Salernitana” University of Salerno Fisciano Italy; ^5^ Neurology Unit, AOU San Giovanni di Dio e Ruggi d'Aragona Salerno Italy; ^6^ Cardiovascular Research Unit, IRCCS Multimedica Milan Italy

**Keywords:** natural killer, progressive supranuclear palsy, VEGF‐A

## Abstract

**Background:**

Emerging evidence implicates neuroinflammation in progressive supranuclear palsy (PSP) pathophysiology, with elevated cyto‐chemokines suggesting natural killer (NK) cell involvement.

**Methods:**

We characterized peripheral NK in PSP (N = 11) versus Parkinson's disease (PD, N = 10) and healthy controls (HC, N = 8) at both immunophenotypic and transcriptional levels.

**Results:**

PSP patients showed significantly reduced CD3‐CD56 + NK frequency (1.35% ± 0.98%) compared with HC (2.96% ± 1.14%) and PD (2.03% ± 0.86%), specifically affecting the immunoregulatory CD56brightCD16−/dim subset. PSP NK cells exhibited elevated CX3CR1 expression, suggesting enhanced migratory capacity toward inflamed tissues. Transcriptomic analysis of primary NK cells revealed 208 DEG with significant enrichment in angiogenesis pathways, particularly vascular endothelial growth factor‐A (VEGF‐A). Plasma VEGF‐A measurements demonstrated a disease‐specific pattern: inverse correlation with severity in PSP versus direct correlation in PD.

**Conclusions:**

These findings identify a potentially disease‐specific transcriptional signature with repercussions on the cyto‐chemokine plasma profile. Further investigation of the NK cell‐mediated immune response in PSP may provide new insights into disease mechanisms and open avenues for immunomodulatory therapeutic strategies. © 2026 The Author(s). *Movement Disorders* published by Wiley Periodicals LLC on behalf of International Parkinson and Movement Disorder Society.

Currently, progressive supranuclear palsy (PSP) diagnosis^1^, ^2^ is based on clinical criteria potentially supported by neuroimaging findings. The search for a diagnostic and prognostic biomarker in cerebrospinal fluid or plasma is ongoing. Although data on the role of neurofilament light chain (NfL) and other biomarkers are encouraging, results are not yet easily applicable to real‐world clinical settings or clinical trials.^3^, ^4^, ^5^

Emerging evidence implicates neuroinflammation in PSP severity and progression.^6^, ^7^ More recently, a comprehensive analysis of the immune signature of the anterior cingulate cortex of PSP patients revealed a peculiar proteomic profile characterized by high levels of CXCL9, CXCL13, IL‐3, and SCF.^8^ Notably, CXCL19 along with CXCL13 are well‐known chemoattractants for activated natural killer (NK) cells. Furthermore, stem cell factor (SCF), together with interleukin‐2 (IL‐2)/IL‐15, enhances NK cell survival and expansion. Similarly, IL‐3 has been reported to maintain lymphoid progenitor development and promote both NK cell and B cell differentiation. Collectively, higher levels of these proteins suggest an association of PSP with peripheral inflammation and particularly with NK cells. A role for NK has been proposed for several neurodegenerative diseases such as PD and amyotrophic lateral sclerosis (ALS).^9^ However, the profile of peripheral immune response and the exact role of NK remain elusive in PSP.

The aim of the present study was to characterize NK subset changes in peripheral blood in PSP compared with both PD patients and controls and to identify a potentially disease‐specific transcriptional signature with repercussions on the cyto‐chemokine plasma profile.

## Patients and Methods

### Patients

The diagnosis and phenotypic categorization of patients were both established by expert clinicians in accordance with current criteria^10^, ^11^, ^12^ as detailed in Supplementary Material.

### RNA Extraction, Sequencing, and Transcriptome Analysis

Total RNA was extracted using the TRIzol™ reagent (Thermo Fisher Scientific) according to the manufacturer's instructions.^13^, ^14^ The obtained reads were aligned on human genome (hg38) with STAR (v2.7.11b),^15^ and expressed transcripts were counted using featureCounts (v2.0.1) and annotated with the gencode v46 primary assembly annotation file. Differential expression analysis was performed using DESeq2 package (v1.46.0)^16^ in R, with default parameters and considering only transcripts that showed a |FC| ≥1.5 and adjusted *P* ≤ 0.05. Gene set enrichment analysis (GSEA)^17^ was conducted using hallmark as the gene set. Circos plots were generated exploiting CIRCOS.^18^

### Statistical Analysis

In all the experiments described, statistical analysis was performed using the GraphPad Prism 10.0 software package for Windows (GraphPad software). Before performing the analysis of variance (ANOVA) and unpaired *t*‐test, data normality was confirmed using both the Shapiro–Wilk test (*P* > 0.05) and the D'Agostino–Pearson omnibus normality test (*P* > 0.05).

## Results

### NK Phenotyping

**FIG. 1:**
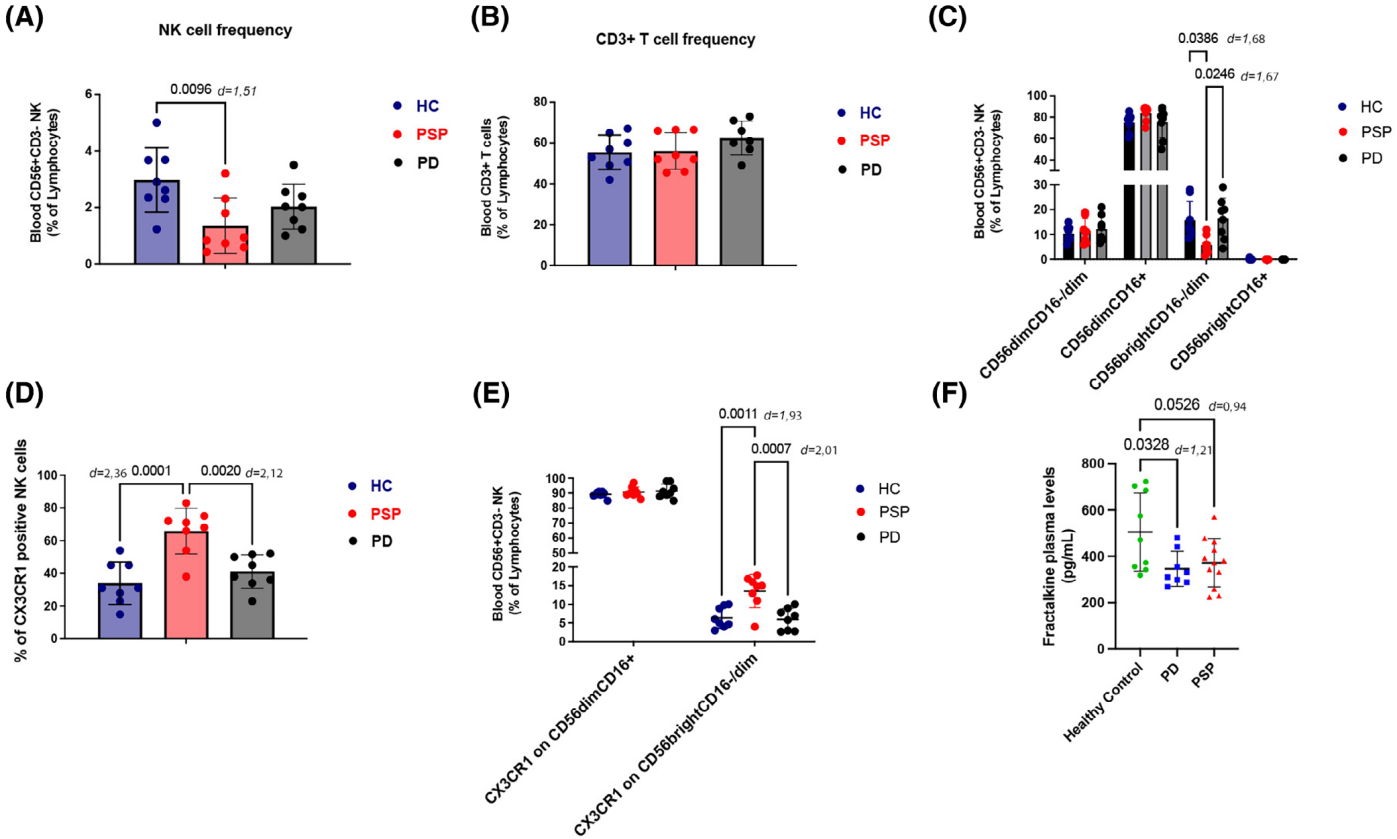
Frequency and phenotype of natural killer (NK) cells in progressive supranuclear palsy (PSP) and Parkinson's disease (PD) patients. (A, B) Cytofluorimetric analysis of CD56 + CD3− NK cell (A) and CD3+ T cell frequency (B) within peripheral blood mononuclear cells (PBMC) gated on lymphocytes in PSP patients (N = 8) and PD patients (N = 8) in comparison with healthy controls (HC, N = 8) in the same experimental conditions. Bar graphs report mean ± standard deviation (SD); statistical significance was determined by one‐way analysis of variance (ANOVA) with Tukey's honest significance test (HSD) post‐hoc test for multiple comparisons. (C) Bar graphs report mean ± SD of the CD56 + CD3− NK cell subsets distribution of CD56^dim^CD16−/dim, CD56^dim^CD16+, CD56^bright^CD16−/dim, and CD56^bright^CD16−/dim NK subsets within PBMC gated on lymphocytes of all PSP and PD patients tested in comparison with HC; statistical significance was determined by two‐way ANOVA followed by Holm–Sidak's post‐hoc test for multiple comparisons. (D, E) Expression of CX3CR1 on total CD56 + CD3− NK cells (D) and selectively on the most abundant subsets of CD56brightCD16−/dim NK cell subsets in comparison with CD56dimCD16+ NK cells from PBMC of all groups tested; statistical significance was determined by one‐way ANOVA with Tukey's HSD post‐hoc test for multiple comparisons (E). Enzyme‐linked immunosorbent assay (ELISA) plasma dosage of CX3CL1/fractalkine circulating levels; statistical significance was determined by one‐way ANOVA with Tukey's HSD post‐hoc test for multiple comparisons (F). *P*‐values are displayed only for pairwise comparisons that are statically significant. Cohen's effect size is reported. [Color figure can be viewed at wileyonlinelibrary.com]

NK phenotyping was conducted on a sample of 8 PSP (85% men, age: 67.5 ± 2.78 years; disease duration: 6.25 ± 1.75 years; 62.5% with Richardson's syndrome), 8 PD (85% men, age: 66 ± 3.5 years; disease duration: 7.11 ± 4.18 years), and 8 healthy controls (HC) (71% men; age: 68.7 ± 7 years) matched for sex, age, and disease duration. Of note, PSP patients showed a lower frequency of CD3‐CD56+ NK cells (1.35% ± 0.98%) with respect to HC (N = 8) (2.96% ± 1.14%, Cohen's *d* = 1.51) and PD patients (2.03% ± 0.86%, *d* = 0.75) (Fig. 1A); in contrast, the percentage of CD3+ T cells did not change across the various groups (Fig. 1B). Examining NK cell subset distribution, which included mainly cytotoxic CD56dim cells and immunoregulatory CD56bright cells, a selective decrease of CD56brightCD16^−/dim^ cells was found in the periphery of PSP (5.75% ± 3.6%, Cohen's *d* = 1.68) but not PD patients (16.3% ± 8.1%, *d* = 0.07) with respect to HC (15.75% ± 7.5%), while other subsets remained unchanged (Fig. 1C). Furthermore, exclusively in PSP patients, NK cells overall (Fig. 1D) and more specifically CD56brightCD16^−/dim^ (Fig. 1E) displayed higher surface levels of CX3CR1 (*P* = 0.0011, *d* = 1.93 vs. HC and *P* = 0.0007, *d* = 2.01 vs. PD). An enzyme‐linked immunosorbent assay (ELISA) assay of the endogenous ligand of this receptor, fractalkine (CX3CL1), showed that its plasma levels were significantly lower in both PD (346.2 ± 75.74 pg/mL, *d* = 1.21) and PSP (372.6 ± 104.31 pg/mL, *d* = 0.94) patients compared with control donors (505 ± 168.37 pg/mL) (Fig. 1F).

### Disease‐Specific Transcriptional Signature

**FIG. 2:**
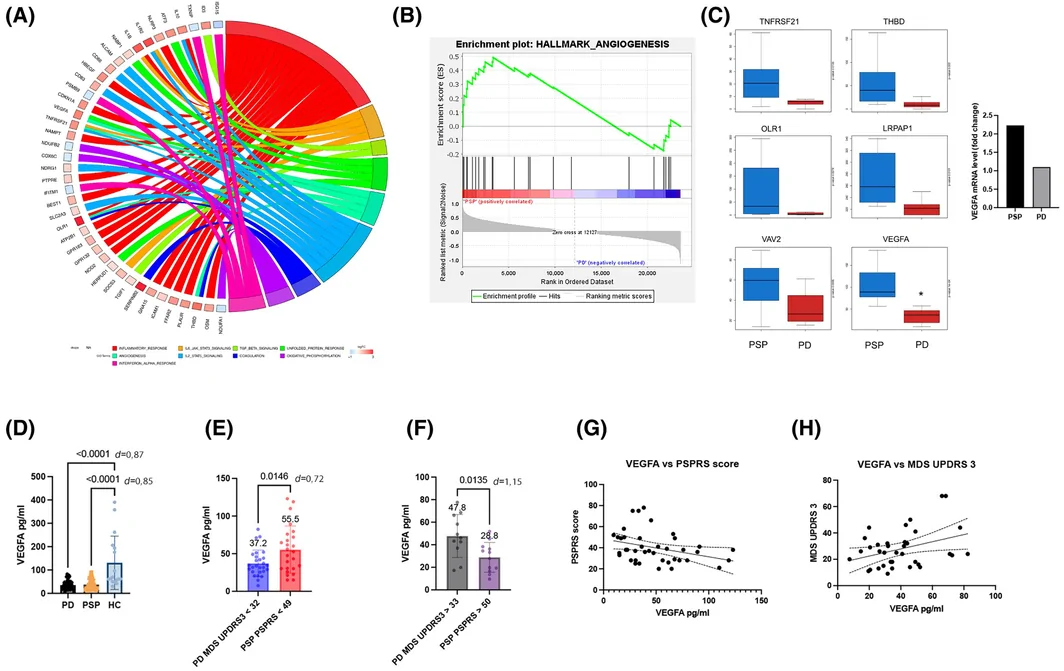
RNA sequencing (RNA‐Seq) analysis from natural killer (NK) cells of progressive supranuclear palsy (PSP) versus Parkinson's disease (PD) and stratifying by relative severity of the disease in groups with high or low levels of vascular endothelial growth factor‐A (VEGF‐A). (A) GO‐plot showing representative pathways involving differentially expressed transcripts in total RNA‐Seq data from NK cells of PSP versus PD. Different pathways are drawn with different colours with their relative genes. LogFC ranking from −1 (blue) to 3 (red) indicates down‐ and up‐regulated genes, respectively. (B) Angiogenesis pathway enriched in gene set enrichment analysis (GSEA) analysis. (C) Boxplots showing the normalized count distribution of genes involved in the angiogenesis pathway enriched in GSEA analysis. The *P*‐value (*< 0.05) is indicated in the right panel. Reverse transcription‐quantitative polymerase chain reaction (RT‐qPCR) displaying VEGF‐A expression level in PSP/PD patients' samples (left panel). (D) Plasma VEGF‐A levels in the study cohorts compared with healthy controls (HC); statistical significance was determined by one‐way analysis of variance (ANOVA) with Tukey's honest significance test (HSD) post‐hoc test for multiple comparisons. (E, F) VEGF‐A levels stratified according to the relative severity of the disease; statistical significance was determined by a two‐tailed unpaired *t*‐test with Welch's correction. (G, H) A correlation analysis between the severity score of both diseases and the VEGF‐A circulating growth factor. Statistical analysis was performed with Prism Graphpad software. Pairwise comparisons statically significant (ANOVA) and Cohen's effect size are reported. [Color figure can be viewed at wileyonlinelibrary.com]

A comprehensive analysis of the transcriptomic profiles of positively selected NK cells was undertaken in a larger sample of PSP (11 patients; 63.6% men; age: 69 ± 4.75 years; disease duration: 5.1 ± 1.66 years; 72.7% with Richardson's syndrome: PSP rating scale: 46.18 ± 15.41) and PD (10 patients; 63.6% men, age: 67 ± 3.5 years; disease duration: 6.11 ± 4.18 years; International Parkinson and Movement Disorder Society‐Unified Parkinson's Disease Rating Scale‐Part III [MDS‐UPDRS‐III]: 21.2 ± 8.65) to identify a disease‐specific transcriptional signature. The differential expression analysis displayed 208 deregulated genes between PSP and PD patients, respectively (135 up‐ and 72 down‐regulated) (Table S1). GSEA was conducted to evaluate pathway involvement. In addition to inflammatory JAK/STAT and transforming growth factor‐beta (TGF‐β) signaling—potentially linked to NK activation^19^—and the unfolded protein response associated with neurodegeneration,^20^, ^21^ one of the most significant enrichments was found in angiogenesis and coagulation‐related categories (Fig. 2A). Among the differentially expressed genes, vascular endothelial growth factor‐A (VEGF‐A) emerged as particularly significant (Fig. 2C). VEGF‐A plays a crucial role in vascular homeostasis and has been directly associated with neurodegeneration and cognitive decline in Alzheimer's disease.^22^ The production of VEGF‐A by NK cells, particularly by the CD56brightCD16‐ subset under hypoxic conditions^23^ or following prolonged TGF‐β exposure,^24^ has been previously documented, suggesting a potential role of NK cells in vascular regulation in neurodegenerative contexts. Further, the preliminary assessment of a different VEGF‐A secretory profile by IL‐2‐activated NK cells from PSP and PD patients (Fig. S1) in cell culture prompted us to measure circulating VEGF‐A.

The measurement of plasma VEGF‐A levels revealed a significant decrease in both PSP and PD patients compared with HC (Fig. 2D). By applying a data‐driven approach, PSP and PD were then stratified into two groups based on disease severity using the median value of the respective rating scale as the cut‐off point in our cohort (PSP with more severe disease PSP rating scale≥50; PSP with less severe disease<49; PD with more severe disease MDS‐UPDRS‐III≥33; PD with less severe disease MDS‐UPDRS‐III<32). Figure 2E–H shows that VEGF‐A presented an inverse correlation with severity of disease in PSP, being lower in more advanced stages. Conversely, VEGF‐A presented a linear correlation with disease severity in PD, being higher in more advanced disease stages.

## Discussion

This study addresses a critical gap in four‐repeat (4R)‐tauopathy immunobiology by providing preliminary evidence on the involvement of the innate NK cells^19^ in the pathophysiology of PSP, identifying specific phenotypic and functional alterations that distinguish this condition from PD and HC. The presence of NK cells in the brain has been described by recent transcriptomic analyses of mouse brain myeloid cells in both healthy and disease states,^25^ implicating their potential role in homeostasis and neurological disorders in the central nervous system (CNS).^26^, ^27^, ^28^, ^29^

Our pilot study demonstrates a reduced frequency of total CD3‐CD56+ NK cells in peripheral blood of PSP patients compared with HC and PD patients. Notably, this decrease is selectively attributable to the CD56brightCD16−/dim subpopulation, known for its immunoregulatory properties and cytokine‐secreting capacity. This may reflect their preferential migration toward sites of neuroinflammation in the CNS, a hypothesis supported by the increased expression of CX3CR1 observed exclusively in PSP patients. Indeed, the increased expression of CX3CR1 in the CD56bright subpopulation, together with the reduction in plasma fractalkine levels observed in both PSP and PD, suggests an active process of cellular recruitment. It is possible that fractalkine is consumed during the chemotaxis process or that its production is altered in the context of neurodegeneration, representing a potential compensatory mechanism attempting to regulate immune cell trafficking to the brain. Nonetheless, given the small sample size, our findings are preliminary and require validation in larger cohorts. Furthermore, future studies should investigate whether the selective reduction of the immunoregulatory CD56bright subset differs across clinical phenotypes. This will help elucidate whether these changes are intrinsic to disease pathology or represent a failed protective immune response. The transcriptomic analysis of NK cells isolated from PSP patients revealed a disease‐specific signature with significant enrichment of the VEGF‐A gene in PSP compared with PD. Strikingly the relationship between VEGF‐A levels and disease severity differed markedly between the two conditions. Although the impact on VEGF‐A plasmatic level is unclear, there is evidence that polymorphisms located within the VEGF promoter represent a susceptibility factor for PSP.^30^, ^31^ As for PD and in line with our data, the presence of oligomeric α‐synuclein results in increased production and release of VEGF‐A produced by astrocyte activation.^32^ Hence, the divergent pattern found in our study may suggest distinct pathophysiological mechanisms underlying vascular dysregulation in PSP versus PD, potentially reflecting different degrees of neuroinflammation, hypoxia, or compensatory angiogenic responses. Indeed, VEGF‐A has a pleiotropic role within the CNS. Besides its classical role in angiogenesis, recent research emphasizes essential non‐vascular functions in neurodevelopment, neuroprotection, and synaptic plasticity.^33^ Investigating the potential role of VEGF‐A in a variety of neurodegenerative diseases spanning movement disorders to dementia should be a key future research direction. Overall, even though future investigations to validate and extend our preliminary findings are necessary, the reduced peripheral NK cell frequency, coupled with increased CX3CR1 expression and altered VEGF‐A dynamics, positions NK cells as potentially important players in PSP pathophysiology. In the context of PSP, the selective reduction of the immunoregulatory CD56bright subset, combined with their heightened migratory potential, raises important questions about whether these cells contribute to disease pathology or represent a failed protective response. The correlation between disease severity and VEGF‐A levels in PSP suggests that as the disease progresses, the capacity of NK cells to maintain vascular homeostasis may become increasingly compromised, potentially contributing to the accelerated neurodegeneration characteristic of PSP compared with PD.

In the future, studies with larger samples and longitudinal evaluation will be necessary to definitively establish causality and temporal relationships between NK cell alterations and disease progression. Additionally, direct assessment of NK cell presence and function within the CNS of PSP patients would provide more definitive evidence of their role in disease pathophysiology. Future activities should also investigate whether the observed NK cell alterations are specific to PSP among atypical parkinsonian syndromes or represent a broader feature of 4R‐tauopathies or frontotemporal lobar degeneration syndromes. To this end it would be interesting to correlate NK cell alterations and the regions of interest primarily affected.

In conclusion, our observations suggest that NK cells may represent both potential biomarkers for disease monitoring and possible therapeutic targets. The distinct relationship between VEGF‐A levels and disease severity in PSP versus PD further underscores the heterogeneity of parkinsonian syndromes and highlights the need for disease‐specific therapeutic approaches.

## Author Roles

(1) Research Project: A. Conception, B. Research Team Design and Coordination, C. Patient Enrolment Coordination, D. Ex Vivo Experiments and Natural Killer Analysis, E. Transcriptomic Analyses, F. Clinical Data Analysis, G. Project Revision; (2) Statistical Analysis: A. Design, B. Execution, C. Review and Critique; (3) Manuscript Preparation: A. Writing of the First Draft, B. Review and Critique, C. Final Approval for Publication; (4). Other: A. Supervision, B. Financial Support.M.P.: 1C, 1F, 3A, 3C.

C.B.: 1D, 3C.

F.M.: 1D, 3C.

V.L.: 1D, 3C.

M.O.: 1F, 1G, 1C, 3C.

R.T.: 1E, 3C.

D.P.: 1E, 3C.

V.M.: 1E, 3C.

M.F.T.: 1F, 1G, 1C, 3C.

P.B.: 1F, 1G, 1C, 3C.

A.A.P.: 3A, 3C.

E.C.: 1B, 1G, 3C, 4A, 4B.

## Financial Disclosures of All Authors (for the Past 12 Months)

M.P. received grants from the Italian Ministry of Health, the Italian Ministry of University, and Fondazione della Società Italiana di Neurologia and speaking honoraria from AbbVie and Zambon.

## Supporting information


**Data S1.** Supporting Information.

Figure S1.

Table S1.

## Data Availability

The data that support the findings of this study are available on request from the corresponding author. The data are not publicly available due to privacy or ethical restrictions.
