# Supplementary material for: Natural Killer Subset Changes and Vascular Endothelial Growth Factor‐A Plasma Profile in Progressive Supranuclear Palsy: The NKscape Study
Source: Mov Disord. 2026 Mar 17;41(8):2201–6. doi: 10.1002/mds.70273 (PMC13518293; doi:10.1002/mds.70273)
Supplement: Supplementary file 1 [file MDS-41-2201-s001.docx]

**Supplementary Methods**

**Patients**

PSP patients were approached from the outpatient clinic of the Centre for Neurodegenerative Diseases (CEMAND), Department of Medicine, Surgery and Dentistry, “Scuola Medica Salernitana”, of the University of Salerno, Italy from September 2023 to September 2025. The diagnosis and phenotypic categorization (ie, Richardson’s syndrome, RS, versus non-Richardson’s syndrome, non-RS) were both established by expert clinicians according with current criteria (2). Inclusion criteria were as follows: (a) age > 40 and < 89 years, (b) ability to walk for at least 5 steps either independently or with a minimum single support (holding to caregiver’s arm or with a walker). The following exclusion criteria were applied: (a) presence of severe sensory deficits (for example, visual or hearing impairment), (b) history of drug abuse, (c) history of severe psychiatric disorders, (d) cerebrovascular diseases or history of traumatic brain injury, (e) Mini-Mental State Examination score < 20. All patients were on a stable medication regimen for at least one month before study entry. Disease severity was evaluated with the PSP rating scale (10). PD patients were approached from the same outpatient clinic and diagnosed according to recent clinical criteria (11). Disease severity was evaluated with the Movement disorder Society-modified Unified Parkinson’s disease rating scale part III (MDS-UPDRS-III) (12). Spouses and caregivers of patients attending the clinic were asked to join the study as Healthy Controls (HC) provided they had no symptoms or signs of any neurologic or psychiatric condition.

**Cell separation, antibodies and flow cytometry and ELISA.**

The peripheral blood mononuclear cell (PBMCs) from patients and healthy donors were extracted from whole blood by Ficoll density gradient (Histopaque®-1077, Sigma–Aldrich, Darmstadt, Germany). After separation, CD56+ NK cells were positively selected from PBMCs by immunomagnetic procedure (Miltenyi Biotec). For the citofluorimetric analysis the following antibodies were used: CD56 (318322) Biolegend, CD3 (130-113-136) Miltenyi Biotec, CX3CR1 (130-105-897) Miltenyi Biotec, CD16 (130-113-393) Miltenyi Biotec. Supernatants from IL-2 stimulated NK cells were assayed for VEGFA secretion through Elabscience Human VEGF-A ELISA Kit. For plasma quantification Human CX3CL1/Fractalkine ELISA Kit (Bio-techne Catalog # DCX310) and VEGFA - Simple Plex Kit Ella instrument Bio-techne were used.

**RNA extraction, sequencing and transcriptome analysis**

Total RNA was extracted using the TRIzol™ reagent (Thermo Fisher Scientific) according to the manufacturer’s instructions. Briefly, 1 mL of TRIzol™ was added to each cell pellet to lyse the cells. Then, 1/10 volume of 1-bromo-3-chloropropane (Sigma-Aldrich) was added as a phase-separating agent. After 10 minutes of incubation, the samples were centrifuged for 15 minutes at 12,000 × *g* at 4 °C. The aqueous phase containing RNA was transferred to a new tube and purified using the RNA Clean & Concentrator kit (Zymo Research) according to the manufacturer’s instructions.
Purified RNAs were eluted in nuclease-free water, and their concentration was determined using a Qubit Fluorometer (Life Technologies). RNA purity and integrity were assessed using NanoDrop 2000c spectrophotometer (Thermo Fisher Scientific) and Agilent 4200 TapeStation system (Agilent Technologies), respectively. Sequencing libraries were prepared from 100 ng of input RNA using the TruSeq Stranded Total RNA kit (Illumina), according to the manufacturer’s protocol, and sequenced at a concentration of 1.3 pM in paired-end mode (2 × 100 bp) on a NovaSeq 6000 platform (Illumina Inc.). Raw reads were pre-processed as previously described (13). In brief, these were demultiplexed using bcl2fastq (v2.20.0.422 Illumina, SCR-015058) and FastQC software (<http://www.bioinformatics.babraham.ac.uk/projects/fastqc/>) was used to assess the reads quality and adapter sequences were removed using cutadapt (v3.3) (14). The obtained reads were aligned on human genome (hg38) with STAR (v2.7.11b) (15), expressed transcripts were counted using featureCounts (v2.0.1) and annotated with the gencode v46 primary assembly annotation file. A transcript was considered as expressed when supported by at least > 10 raw counts. Differential expression analysis was performed using DESeq2 package (v1.46.0) (16) in R, with default parameters and considering only transcripts that showed a |FC| ≥1.5 and adjusted p-value ≤ 0.05. The Gene Set Enrichment Analysis was conducted with GSEA (17) using hallmark as gene set. Circos plot was generated exploiting CIRCOS (18).

**Statistical analysis**

In all experiments shown, statistical analysis was performed using the GraphPad Prism 10.0 software package for Windows (GraphPad software). For each type of assay or phenotypic analysis, the data obtained from multiple experiments were calculated as mean±SD and analyzed for statistical significance using appropriate tests. In the analysis of variance (ANOVA) for multiple comparisons, p-values<0,05 were considered significant.
